# Implantation of Neural Probes in the Brain Elicits Oxidative Stress

**DOI:** 10.3389/fbioe.2018.00009

**Published:** 2018-02-12

**Authors:** Evon S. Ereifej, Griffin M. Rial, John K. Hermann, Cara S. Smith, Seth M. Meade, Jacob M. Rayyan, Keying Chen, He Feng, Jeffrey R. Capadona

**Affiliations:** ^1^Department of Biomedical Engineering, Case Western Reserve University, Cleveland, OH, United States; ^2^Advanced Platform Technology Center, Louis Stokes Cleveland Department of Veterans Affairs Medical Center, Cleveland, OH, United States

**Keywords:** oxidative stress, intracortical microelectrodes, gene expression, histology, brain

## Abstract

Clinical implantation of intracortical microelectrodes has been hindered, at least in part, by the perpetual inflammatory response occurring after device implantation. The neuroinflammatory response observed after device implantation has been correlated to oxidative stress that occurs due to neurological injury and disease. However, there has yet to be a definitive link of oxidative stress to intracortical microelectrode implantation. Thus, the objective of this study is to give direct evidence of oxidative stress following intracortical microelectrode implantation. This study also aims to identify potential molecular targets to attenuate oxidative stress observed postimplantation. Here, we implanted adult rats with silicon non-functional microelectrode probes for 4 weeks and compared the oxidative stress response to no surgery controls through postmortem gene expression analysis and qualitative histological observation of oxidative stress markers. Gene expression analysis results at 4 weeks postimplantation indicated that EH domain-containing 2, prion protein gene (Prnp), and Stearoyl-Coenzyme A desaturase 1 (Scd1) were all significantly higher for animals implanted with intracortical microelectrode probes compared to no surgery control animals. To the contrary, NADPH oxidase activator 1 (Noxa1) relative gene expression was significantly lower for implanted animals compared to no surgery control animals. Histological observation of oxidative stress showed an increased expression of oxidized proteins, lipids, and nucleic acids concentrated around the implant site. Collectively, our results reveal there is a presence of oxidative stress following intracortical microelectrode implantation compared to no surgery controls. Further investigation targeting these specific oxidative stress linked genes could be beneficial to understanding potential mechanisms and downstream therapeutics that can be utilized to reduce oxidative stress-mediated damage following microelectrode implantation.

## Introduction

Intracortical microelectrodes were initially designed as a neuroscience tool to allow researchers the ability to investigate and understand how the nervous system works (Renshaw et al., 1940; Grundfest and Campbell, 1942; Grundfest et al., 1950). In addition to their role as a research tool, intracortical microelectrodes have the ability to treat patients with a wide range of neurological injuries and degenerative diseases, either directly through clinical implantation or indirectly by giving researchers a tool to better understand these diseases. For example, intracortical microelectrodes were used recently to allow patients with amyotrophic lateral sclerosis (ALS) to use their thoughts to control virtual neural cursors on the computer screen (Gilja et al., 2015). Over the past two decades, brain computer interfaces involving intracortical microelectrodes have entered clinical trials for patients with motor deficits, such as spinal cord injuries and ALS (Gilja et al., 2015; Schroeder and Chestek, 2016; Ajiboye et al., 2017). Unfortunately, recording quality of microelectrodes decreases within weeks and diminishes within a few years due to the complex inflammatory response observed after electrode implantation (Chestek et al., 2011; Jorfi et al., 2014; Kozai et al., 2015).

**Figure 1:**
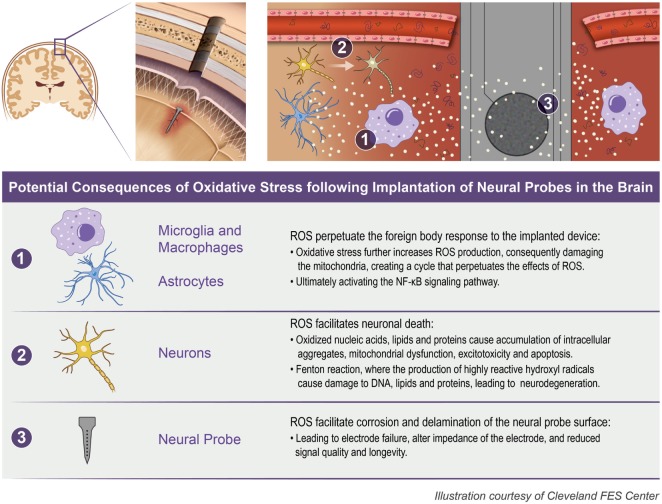
Oxidative stress following neural probe implantation. The implantation of neural probes leads to the overproduction of reactive oxygen species (ROS) which can consequently (1) perpetuate the foreign body response, (2) facilitate neuronal death, and (3) facilitate corrosion and delamination of the microelectrode surface.

The initial insertion of intracortical microelectrodes results in an injury of the brain tissue, eliciting a chain reaction of chemical and biological events that contributes to the ultimate failure of the device to record action potentials for local neurons (Polikov et al., 2005; Potter et al., 2012a; Kozai et al., 2015). One mechanism that has been suggested to play a key role in the failure of microelectrodes is oxidative stress at the microelectrode–tissue interface (McConnell, 2008; Saxena et al., 2013; Potter et al., 2014; Potter-Baker and Capadona, 2015; Potter-Baker et al., 2015; Nguyen et al., 2016). Specifically, the presence of oxidative stress can (1) directly facilitate neuronal cell death (2) perpetuate the foreign body response to the implanted device, and (3) facilitate corrosion and delamination of the microelectrode surface (Prasad et al., 2012, 2014; Potter et al., 2013; Takmakov et al., 2015). Figure 1 illustrates the potential consequences from oxidative stress that can occur following the implantation of neural probes in the brain.

The breaching of the blood–brain barrier results in an infiltration of neurotoxic factors and pro-inflammatory cells which lead to neuronal degeneration and death (Potter et al., 2013; Saxena et al., 2013). Pro-inflammatory cells (activated microglia, macrophages, and astrocytes) remain around the implant site for the duration of implantation (McConnell et al., 2009; Ravikumar et al., 2014; Nguyen et al., 2016). Furthermore, it is understood that these pro-inflammatory cells release free radicals, reactive oxygen species (ROS), and reactive nitrogen species when activated (Streit et al., 1999; Abbott et al., 2006; Kettenmann et al., 2011). The release of reactive species and radicals around implanted intracortical microelectrodes can lead to oxidation of the electrode surface, and as a result, the corrosive breakdown of the material (Schmitt et al., 1999; Takmakov et al., 2015). For example, Prasad et al. (2014) demonstrated the accumulation of ferritin, indicative of perpetuating oxidative stress, around implanted functional microelectrodes 10 weeks after implantation, and suggested a correlation to the corrosion of both insulating and conductive microelectrode material components. McConnell et al. (2009) reported that implantation of microelectrodes could result in the accumulation of hemosiderin-laden macrophages, indicating that the implant site was hemorrhagic and speculated to be a byproduct of oxidative stress, as early as 2 weeks and up to 16 weeks post-microelectrode implantation. Additionally, Takmakov et al. (2015) showed that ROS, released in their reactive accelerated aging (RAA) *in vitro* system, created structural damage to microelectrode arrays thereby altering the electrical properties *via* decreased electrode impedance. The decline in impedance in their *in vitro* RAA system, which simulated 6 months *in vivo*, was reported to be consistent with published reports on *in vivo* impedance changes (Takmakov et al., 2015).

The brain is highly susceptible to oxidative stress due to its biochemical composition, specifically unsaturated lipids, which are targeted for oxidative modification and lipid peroxidation (Gallego et al., 2011). Furthermore, due to the brain’s high oxygen requirement (20% of the total oxygen intake is used), it has an increased risk of peroxidation (Gallego et al., 2011). Specifically, neurons are the most vulnerable cell to oxidative damage, due to their high content of methyl ions and low antioxidant activity (Floyd and Carney, 1992; Gallego et al., 2011). When subjected to a continuous state of oxidative stress, neurons result in severe damage to their cellular constituents including proteins, DNA, and lipids (Dawson and Dawson, 1996; Gallego et al., 2011). The pathology and molecular biomarkers for diseases such as Alzheimer’s and Parkinson’s disease include neurodegeneration and neuronal cell death, which have been linked to the abnormal cellular proteins and lipids formed due to ROS accumulation (Smith et al., 2000; Emerit et al., 2004; Lin and Beal, 2006; Gallego et al., 2011). Notably, our lab has shown the use of antioxidants, either locally or systemically, results in higher densities of neuronal nuclei and more viable neurons at the intracortical microelectrode/tissue interface (Potter et al., 2013, 2014; Jorfi et al., 2014; Potter-Baker et al., 2014; Nguyen et al., 2016).

The above literature review established that there have been many studies which suggest oxidative stress as a key component of the failure mechanism of intracortical microelectrodes. However, a definitive link has yet to be determined. Given the potential role oxidative stress events play in the failure of intracortical microelectrodes, it is crucial to elucidate and identify the specific cellular and molecular oxidative stress factors involved after intracortical microelectrode implantation. While most previous studies, including our own lab, have focused on the histological analysis of neuroinflammation, the use of gene expression has been shown to be more sensitive than histological analysis—providing more insight into the phenotype of the cells (Karumbaiah et al., 2013; Ereifej et al., 2017). Information with respect to inflammatory and non-inflammatory cell phenotype may more directly facilitate intervention strategies that are clinically translatable if intervention strategies are more specific, minimizing unintentional side effects of broader spectrum therapeutics. Thus, the goal of this study is to give direct evidence of oxidative stress following intracortical microelectrode implantation using gene expression analysis and histological approaches. Prior to this study, we hypothesized that there is an increased presence of oxidative stress markers following intracortical microelectrode probe implantation. To evaluate our hypothesis, we implanted adult rats with silicon non-functional microelectrode probes for four weeks and compared the oxidative stress response to no surgery sham controls. To assess the cellular and molecular oxidative stress response to intracortical microelectrode implantation, we quantified oxidative stress markers through postmortem gene expression analysis and qualitatively observed the presence of oxidative stress markers surrounding the implant though histological staining.

## Materials and Methods

### Neural Probe Implantation Procedure

All animal procedures were approved by the Institutional Animal Care and Use Committee at the Louis Stokes Cleveland Department of Veterans Affairs Medical Center. A total of eight adult (8–10 weeks old, ~225 g) male Sprague Dawley rats were used in this study. Four of the rats were implanted with neural probes in the sensory cortex while the other four were used as no surgery sham controls. Genomic analysis was performed on the same animals used for histological analysis in this study. Similar to previous surgical procedures published by this lab, each animal was anesthetized to the surgical plane in an isoflurane chamber (3.5% in 1.5 L/min O_2_) for 4 min (Ereifej et al., 2017; Goss-Varley et al., 2017). Following which, isoflurane was administered through a nose cone at 2.5% in order to shave the incision site and deliver a subcutaneous injection of Marcaine. Subcutaneous Carprofen (5 mg/kg) and Cefazolin (25 mg/kg) injections were given for analgesia and antibiotics, respectively. The rat was then mounted to a stereotaxic frame connected to a nose cone flowing 1–2.5% isoflurane to maintain anesthesia throughout the surgery. Seven alternating cotton tipped applicators of chlorhexidine gluconate and isopropanol were used to sterilize the surgical site. Body temperature was maintained *via* a circulating water pad and vitals (body temperature, heart and respiratory rate, and oxygen levels) were monitored using a heart rate and blood–oxygen measurement system (MouseSTAT^®^ Pulse Oximeter & Heart Rate Monitor, Kent Scientific Corp., Torrington, CT, USA).

The surgery began with an incision down the midline of the head and retraction of the skin to view the skull. The periosteum was cleaned off of the skull with a cotton swab applicator, followed by dehydration of the skull using hydrogen peroxide, and application of Vetbond, an animal tissue adhesive, to prime the skull. A sterile ruler and forceps were used to mark the area to be drilled, 2 mm lateral to midline, 3 mm posterior to bregma (sensory cortex). The dura was carefully reflected using a 45° angle dura pick to expose the brain. The implant was inserted manually using forceps. The surgery site was covered with an insulating silicone elastomer, Kwik-Cast (World Precision Instruments, Sarasota, FL), followed by Fusio and Flow-it ALC (Pentron Clinical, Wallingford, CT) UV-cured dental cement to build a stable headcap covering the entire implant. The skin was sutured shut with 5-0 monofilament polypropylene suture (Henry Schein, Melville, NY, USA), and antibiotic ointment was applied to the suture path. Analgesia and antibiotics were administered for 3 days postoperatively.

### Tissue Processing

Animals were anesthetized by intraperitoneal injections of ketamine (160 mg/kg) and xylazine (20 mg/kg) at 4 weeks postimplantation, as a predetermined end point. Animals were perfused with 1× phosphate buffer saline (PBS, Invitrogen, Carlsbad, CA, USA) to clear the blood, followed by 30% sucrose (Sigma, St. Louis, MO, USA) in 1× PBS to cryoprotect the tissue. The brain was removed carefully from the skull and the electrode was explanted. The brain was then frozen in optimal cutting temperature compound (OCT, Tissue Tek, Torrance, CA, USA) on dry ice and stored at −80°C for cryosectioning.

The cryostat, blades, and slides were decontaminated of RNase enzymes using RNaseZap^®^ (Thermo Fisher Scientific, Waltham, MA, USA). Brains were sliced transversely at 20 µm thick slices and mounted onto either glass slides for staining or Leica FrameSlides PEN-Membrane 4.0 µm (Leica, Wetzlar, Germany) slides for Laser Capture Microdissection and downstream genetic analysis. Slides were then stored at −80°C until LCM or immunohistochemical labeling.

### Laser Capture Microdissection

To prepare for LCM, the slides were removed from −80°C storage and immediately submerged in the following ethanol series: 95% (30 s), 70% (30 s), and 50% (30 s). There were 18 tissue slices per animal used for LCM tissue collection. The tissue was stained with Cresyl Violet (in 50% ethanol), followed by a dehydration series according to the manufacturer’s protocol (AM1935, Ambion, Waltham, MA, USA). Following the dehydration, the tissue was immersed in xylene for 5 min and then air-dried for 5 min. Slides were transferred to an RNase contamination-free Leica LMD7000 microdissection system. The LCM microscope and Leica software was used to identify the implant sites in the surgery tissue, and the respective location in the sham tissue based on Cresyl Violet staining. A 500 µm radius circle was centered on the site of the implant (or sham site), and the tissue was laser cut. The cut tissue pieces were immediately collected in 500 µL tubes containing Qiazol (Qiagen, Valencia, CA, USA), an RNA extraction lysis buffer. Throughout the process, the microdissected tissue samples were preserved on ice. RNA was extracted and purified the same day as collection and stored at −80°C for further processing.

### Real-time Polymerase Chain Reaction

RNA was purified using RNeasy Micro Kit (Qiagen, Valencia, CA, USA) in accordance with the manufacturer’s protocol. The purity and concentration of the RNA was measured using a NanoDrop apparatus measuring the ratio between the 260 and 280 nm wavelengths (Thermo Fisher Scientific, Waltham, MA, USA). Reverse transcriptase converted the mRNA to a cDNA template using random primers and a thermal cycle (GeneAmp PCR System 9700, Applied Biosystems, Foster City, CA, USA) following the manufacturer’s protocol (Qiagen RT2 Profiler, Qiagen, Valencia, CA, USA). PCR analysis was conducted using cDNA equivalent to 40 ng of total RNA used. Oxidative Stress RT^2^ Profiler PCR Arrays (330231; Qiagen, Valencia, CA, USA) containing 84 genes involved in the oxidative stress pathway were utilized. The PCR Arrays contained positive PCR controls, reverse transcriptase controls, genomic DNA contamination controls as well as five endogenous controls, actin beta, beta-2 microglobulin (B2M), hypoxanthine phosphoribosyl transferase 1, lactate dehydrogenase A, and ribosomal protein. For our analysis, the B2M was utilized as the endogenous control. SYBR green (Qiagen, Valencia, CA, USA) was used as the fluorescence tag. cDNA templates along with the master mix were read in a 96-well optical plate. The instrument used for the measurement was a 7900HT Real-Time PCR system (Applied Biosystems) running the following protocol: (1) hold 95°C for 10 min and (2) 40 cycles at 95°C for 15 s and 60°C for 1 min. Melt curves for each gene were ran and evaluated to verify proper runs running the following: (1) hold 95°C for 15 s, (2) hold 60°C for 15 s, and (3) hold 95°C for 15 s. Using the SDS 2.3 software (Applied Biosystems, Foster City, CA, USA) the threshold cycle (Ct) values for each sample and primer pair were calculate. The delta (Δ) Ct method was utilized to calculate the relative gene expression fold change (R) (Livak and Schmittgen, 2001; Schmittgen and Livak, 2008).

The following equations were used:

ΔCt = Ct  (Gene of Interest)  −  Ct  (B2M)

$$R\;=\;{\text{2}}^{\Delta \text{Ct}}$$

### Histology

In order to determine the relationship between neural probe implantation and oxidative stress, immunohistochemistry (IHC) of the peroxidase-anti-peroxidase staining method was used with 3'-3'-diaminobenzidine (DAB; Dako) as a chromogen. Staining was employed to analyze the presence of oxidized nucleic acids (8-hydroxydeoxyguanosine), lipids (hydroxynonenal), and proteins [nitrotyrosine (NT)]. In addition to colorimetric DAB staining, adjacent tissue slices were fluorescently stained for glial fibrillary acidic protein (GFAP) to accurately define the region of implantation by identifying the location of the glial scar surrounding the implant (Potter et al., 2012a). Histology controls for colorimetric DAB staining were no surgery sham controls.

To prepare tissue for IHC staining, previously established protocols were followed (Potter et al., 2013; Nguyen et al., 2014; Ereifej et al., 2017). Briefly, tissue was first equilibrated to room temperature (RT) in a humidity chamber. OCT was removed with three consecutive PBS washes. Each wash consisted of a gentle application of PBS to tissue followed by a 5-min incubation prior to beginning the next wash. Following OCT removal, tissue was fixed with 4% formaldehyde for 10 min at RT.

#### Fluorescent Staining

Following fixation, tissue was rinsed, rehydrated, and permeabilized with PBS containing 0.1% Triton-X (PBS-T). Tissue was then blocked for 1 h with goat serum blocking buffer [4% v/v serum (Invitrogen, Carlsbad, CA, USA), 0.3% v/v Triton-X 100, 0.1% w/v sodium azide (Sigma)]. Next, astrocytic scarring was detected *via* rabbit anti-GFAP (1:500, Dako) for astrocytes. Primary antibodies were incubated for 18 h at 4°C. Following primary antibody incubation, tissue was washed six times for 5 min each with PBS-T. Next, AlexaFlour conjugated antibodies (1:1,000) were incubated for 2 h at RT. DAPI (4′,6-diamidino-2-phenylindole) was included in this incubation to counterstain all cell nuclei. Following incubation, tissue was again washed six times for 5 min each with PBS-T, followed with a 10 min 0.5 mM copper sulfate solution (50 mM Ammonium Acetate, pH 5.0; Sigma) to reduce tissue autofluorescence (Potter et al., 2012b). Samples were finally rinsed with deionized water and mounted with Fluoromount-G (Southern Biotech).

#### Colorimetric Staining

For oxidative stress immunostaining, previously published protocols were followed (Lee et al., 2012). Following fixation (described above), tissue samples were incubated with 3% hydrogen peroxide in methanol for 30 min, to quench inherent peroxidase activity. Next, tissue was rinsed and rehydrated with Tris buffered saline (50 mM Tris, 150 mM NaCl, pH = 7.6, TBS) for 10 min. Following which, tissue was blocked with 10% normal goat serum (NGS, Abcam) in TBS for 30 min and rinsed several times with 1% NGS in TBS. After blocking and rinsing, primary antibodies diluted in 1% NGS were added to the slides. Tissue slices were incubated with antibodies for 2 h at 37°C in a humidity chamber. Antibodies and their corresponding concentrations are listed in Table 1. Following tissue incubation with primary antibodies, tissue was rinsed with 1% NGS, blocked for 10 min with 10% NGS, and rinsed again with 1% NGS. Following this rinse, tissue was incubated with species-specific secondary antibodies (EMD Millipore, Burlington, MA, USA) at RT for 30 min. After incubation with secondary antibodies, tissue was again rinsed several times with 1% NGS in TBS. Next, tissue was incubated with species-specific peroxidase anti-peroxidase (Immunogen) complex at RT for 1 h. Slides were then rinsed with Tris buffer and developed for approximately 5 min with the chomogen DAB (Dako, Santa Clara, CA, USA). Prior to mounting, slides were incubated for 10 min each in the following solutions in succession: 70% ethanol, 95% ethanol, 100% ethanol, and Xylene II. Coverslips were then used to mount the slides using permount. Slides were dried overnight on a warm hot plate at ~ 30°C.

**Table 1:** Histological markers for oxidative stress.

Primary antibody	Oxidative stress marker	Supplier	Species	Dilution
Anti-nitrotyrosine	Oxidized proteins	Cayman Chemical [10189540]	Rabbit	1:500
Anti-8-hydroxydeoxyguanosine	Oxidized nucleic acids	Abcam (15A3) [ab62623]	Mouse	1:500
Anti-hydroxynonenal	Oxidized lipids	Alpha Diagnostics [HNE11-S]	Rabbit	1:3,000

### Imaging

All slides were imaged under 10× magnification using a Carl Zeiss AxioObserver Z1 (Zeiss, Inc.) Fluorescently labeled tissue was imaged utilizing an AxioCam MRm monochrome camera (Zeiss, Inc.). DAB labeled tissue was imaged using an AxioCam ERc5 color camera (Zeiss, Inc.). In order to capture the entire area of implantation, the Mosaix module was used to stitch together a 4 × 4 tile image. Images shown have been enhanced to improve visual representation.

### Statistical Analysis

For statistical analysis of gene expression, *t*-tests in Minitab 16 (Minitab Inc., State College, PA, USA) were performed. All the RNA from one animal was pooled and analyzed as an independent sample. Significance was defined as *p* < 0.05.

Sample size analysis was based upon data observed for Ercc6, Ptgs2 (Cox2), Sod3, and Srxn1 relative gene expression. A power analysis using a two-tailed *t*-test was used to determine the number of animals required to determine statistical significance with a 95% confidence and power of 0.80. Pooled standard deviation of 6.35 for Ercc6, 7.42 for Ptgs2 (Cox2), 7.00 for Sod3, and 5.10 for Srxn1 relative gene expression, and a difference of means between no surgery control and surgery groups of 9.70 for Ercc6, 10.45 for Ptgs2 (Cox2), 10.03 for Sod3, and 8.52 for Srxn1 relative gene expression were assumed.

## Results

### Oxidative Stress Gene Expression after Electrode Implantation

**Figure 2:**
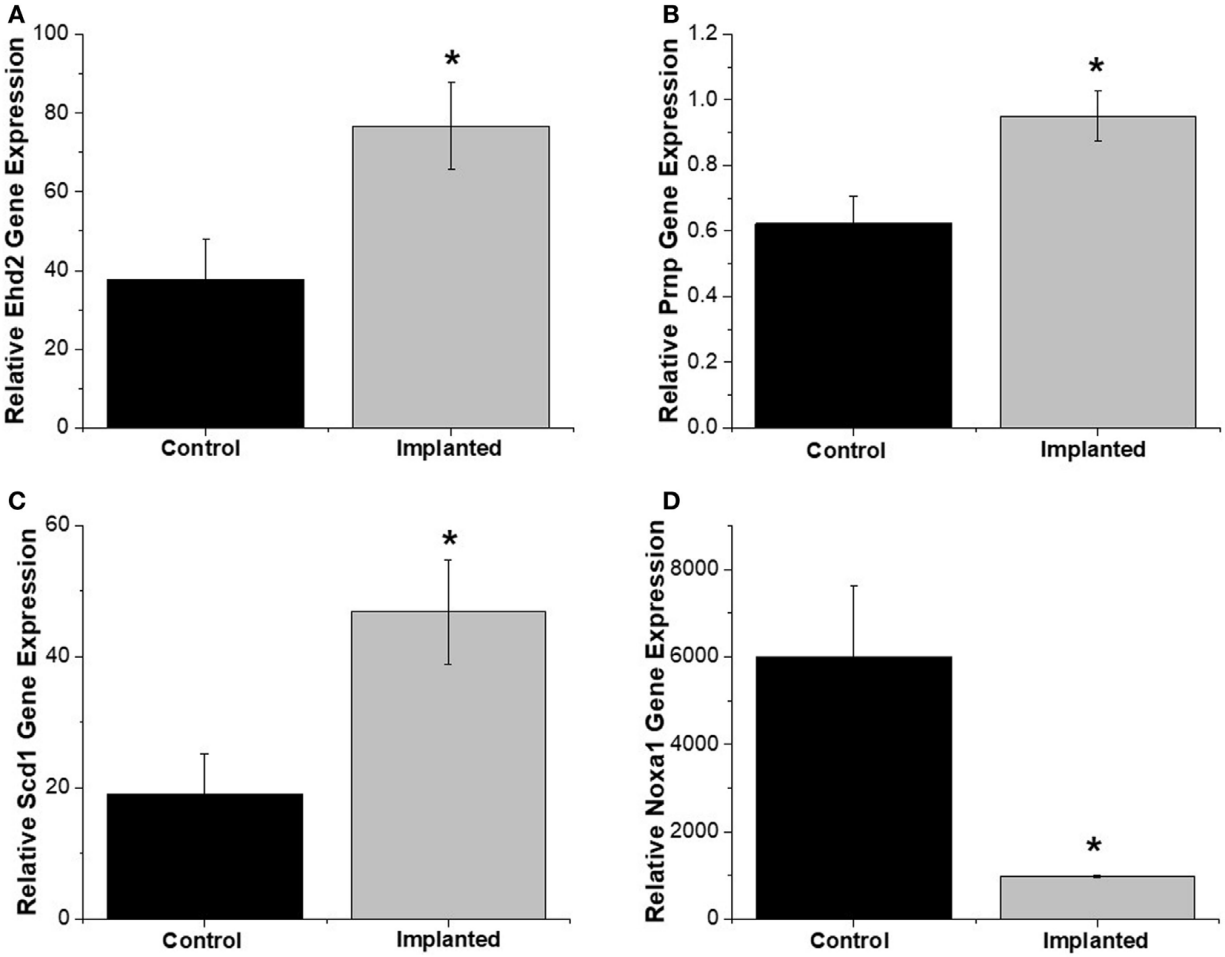
Oxidative stress relative gene expression. Relative gene expression from tissue around implanted animals were quantitatively compared to no surgery control animals. **(A)** EH domain-containing 2 (Ehd2), **(B)** Prnp, and **(C)** Scd1 relative gene expression were significantly higher for implanted animals compared to no surgery controls. **(D)** Noxa1 relative gene expression was significantly lower in implanted animals compared to no surgery controls. * denotes *p* < 0.05.

Gene expression analysis was performed on both implanted and no surgery control animals in order to better understand the molecular markers involved in the oxidative stress pathway occurring after intracortical microelectrode implantation. The use of gene expression has been shown to be more sensitive than histological analysis, while also providing more insight into the phenotype of the cells (Karumbaiah et al., 2013; Ereifej et al., 2017). Therefore, RT-PCR arrays for oxidative stress containing 84 distinct genes of interest involved in oxidative stress pathways were utilized for this study. The array was comprised of antioxidant genes, genes involved in the metabolism of ROS, and oxygen transporters. Of the 84 genes analyzed in the array, there were four genes that revealed statistically significant differences between the surgery and sham animals (Table 2): EH domain-containing 2 (Ehd2), prion protein gene (Prnp), Stearoyl-Coenzyme A desaturase 1 (Scd1), and Nicotinamide adenine dinucleotide phosphate oxidase activator 1 (Noxa1). Specifically, at 4 weeks postimplantation, Ehd2, Prnp, and Scd1 relative gene expression were all significantly higher (*p* < 0.05) from animals implanted with intracortical microelectrode probes compared to no surgery control animals (Figures 2A–C). To the contrary, Noxa1 relative gene expression was significantly lower (*p* < 0.05) from implanted animals compared to no surgery control animals (Figure 2D). Ehd2 gene encodes for the EH domain proteins, found on the plasma membrane, which function in both endocytosis and signal transduction pathways (Pohl et al., 2000). Prnp encodes for the membrane protein, cellular prion protein, a glycosylphosphatidylinositol anchored glycoprotein, which is highly expressed in the brain (Ding et al., 2013). Misfolding of the prion protein has been linked to several neurodegenerative diseases including Alzheimer’s disease and Parkinson’s disease (Wemheuer et al., 2017). Scd1 is a key regulator of lipid metabolism (Ntambi and Miyazaki, 2003; Flowers and Ntambi, 2008; Igal, 2010). The human Scd1 gene is anchored in the membrane of the endoplasmic reticulum and is ubiquitously expressed, with highest levels in brain, liver, heart, and lung (Igal, 2010; Liu et al., 2011). Noxa1 is the gene that encodes and regulates the protein NADPH oxidase (NOX1), which is an enzyme that catalyzes the generation of ROS (Ma et al., 2017). Noxa1 has been reported to be in the blood vessels, neurons, astrocytes, and microglia and in the hippocampus of the brain (Ago et al., 2005; Brown and Griendling, 2009; Choi et al., 2014; Ma et al., 2017).

**Table 2:** Oxidative stress relative gene expression.

Gene name	Control mean	Control SOM	Implant mean	Implant SOM	*p*-Value
**Reactive oxygen species (ROS) metabolism—oxidative stress responsive genes**					
Amyotrophic lateral sclerosis 2 (juvenile) homolog (human)	24.44	2.13	33.06	5.01	0.16
Apolipoprotein E	0.43	0.13	0.51	0.17	0.72
Chemokine (C-C motif) ligand 5	915.56	500.06	748.19	428.07	0.81
24-dehydrocholesterol reductase	17.40	6.57	21.66	5.09	0.65
Dual oxidase 2	550.01	96.50	1009.09	392.53	0.30
Excision repair cross-complementing rodent repair deficiency, complementation group 2	24.90	5.08	36.84	8.24	0.26

Excision repair cross-complementation group 6	13.15	3.27	22.85	3.08	0.07

Ferritin, heavy polypeptide 1	0.11	0.01	0.13	0.01	0.33
Glutamate cysteine ligase, catalytic subunit	7.24	1.89	10.21	1.01	0.22
Glutamate cysteine ligase, modifier subunit	9.30	2.40	13.16	3.16	0.37
Heme oxygenase (decycling) 1	111.85	29.32	79.73	17.77	0.38
Heat shock 70 kD protein 1A	3,366.68	969.35	1936.07	720.94	0.34
Isocitrate dehydrogenase 1 (NADP+), soluble	6.59	0.62	6.52	0.58	0.94
Keratin 1	573.10	x	1924.74	334.64	x
NAD(P)H dehydrogenase, quinone 1	36.97	13.44	36.43	13.26	0.98
Nudix (nucleoside diphosphate linked moiety X)-type motif 1	70.06	14.92	94.44	12.05	0.25
Parkinson disease (autosomal recessive, early onset) 7	1.31	0.12	1.35	0.29	0.89

Prion protein	0.62	0.08	0.95	0.08	0.03

Proteasome (prosome, macropain) subunit, beta type 5	0.97	0.05	1.05	0.17	0.68
Selenoprotein P, plasma, 1	0.91	0.09	0.92	0.07	0.94
Sequestosome 1	2.46	0.21	6.95	3.05	0.19
Thyroid peroxidase	6,405.17	2,319.58	6,092.34	1,814.30	0.94
Thioredoxin 1	1.51	0.15	1.84	0.40	0.47
Thioredoxin interacting protein	28.27	5.63	21.05	4.72	0.36
Uncoupling protein 3 (mitochondrial, proton carrier)	1,586.09	542.11	10,164.09	8,182.48	0.42
**ROS metabolism—superoxide dismutases (SOD)**					
Albumin	33.67	12.19	81.13	22.96	0.12
Glutathione reductase	6.91	1.97	8.56	0.49	0.45
Superoxide dismutase 1, soluble	0.73	0.20	0.99	0.15	0.33
Superoxide dismutase 2, mitochondrial	0.98	0.13	1.31	0.16	0.16

Superoxide dismutase 3, extracellular	15.26	4.33	25.29	2.41	0.09

Sulfiredoxin 1 homolog (*S. cerevisiae*)	11.75	2.77	20.27	2.31	0.06

Thioredoxin reductase 1	19.01	3.06	29.41	8.80	0.31
Thioredoxin reductase 2	33.65	7.07	50.25	7.73	0.16
**ROS metabolism—other superoxide metabolism genes**					
Copper chaperone for superoxide dismutase	15.13	3.89	15.88	4.10	0.90
Cytochrome b-245, alpha polypeptide	234.40	185.93	31.57	18.76	0.32
Neutrophil cytosolic factor 1	183.73	48.07	92.01	27.49	0.15
Neutrophil cytosolic factor 2	235.79	67.46	175.14	49.97	0.50
Nitric oxide synthase 2, inducible	2,495.62	1,381.28	1,809.22	607.35	0.67
NADPH oxidase 4	3,975.34	1,908.32	9,734.48	5,244.35	0.42
NADPH oxidase activator 1	5,995.30	1,148.75	970.92	26.61	0.03

NADPH oxidase organizer 1	3,042.75	1,164.68	7,658.91	1,728.05	0.10

Stearoyl-Coenzyme A desaturase 1	19.14	6.09	46.84	7.98	0.03

Uncoupling protein 2 (mitochondrial, proton carrier)	16.09	4.72	19.45	2.05	0.54
**ROS metabolism—other ROS Metabolism Genes**					
Aldehyde oxidase 1	108.03	38.01	420.53	220.47	0.21
Flavin containing monooxygenase 2	552.62	221.46	859.12	366.67	0.55
**Antioxidants—peroxiredoxins (TPx)**					

EH domain-containing 2	37.82	10.29	76.64	11.07	0.04

Peroxiredoxin 1	1.81	0.32	2.13	0.21	0.44
Peroxiredoxin 2	1.30	0.13	1.21	0.13	0.64
Peroxiredoxin 3	4.18	0.82	5.19	1.18	0.51
Peroxiredoxin 4	10.55	2.97	13.29	1.74	0.46
Peroxiredoxin 5	2.14	0.30	1.92	0.38	0.70
Peroxiredoxin 6	2.38	0.39	2.00	0.29	0.47
**Antioxidants—glutathione peroxidases (GPx)**					
Glutathione peroxidase 1	3.24	0.71	3.69	0.39	0.60
Glutathione peroxidase 2	571.09	150.38	1201.89	602.13	0.35
Glutathione peroxidase 3	26.34	7.77	36.14	7.32	0.39
Glutathione peroxidase 4	0.72	0.28	1.07	0.08	0.28
Glutathione peroxidase 5	15,244.16	4,227.83	25,865.02	12,424.55	0.45
Glutathione peroxidase 6	293,809.24	208,228.14	406,834.19	313,034.87	0.80
Glutathione peroxidase 7	57.77	10.30	51.19	12.75	0.70
Glutathione S-transferase kappa 1	11.65	2.82	11.14	2.65	0.90
Glutathione S-transferase pi 1	3.53	0.20	3.09	0.71	0.57
**Antioxidants—other peroxidases**					
Adenomatous polyposis coli	1.20	0.30	1.64	0.30	0.34
Catalase	8.36	2.02	9.77	1.46	0.59
Cathepsin B	0.92	0.17	1.00	0.11	0.71
Dual oxidase 1	63,887.16	46,458.48	103,022.18	68,166.72	0.72
Eosinophil peroxidase	531.06	183.09	682.10	85.03	0.49
Lactoperoxidase	2,325.71	1,604.08	521,565.32	366,991.43	0.18
Myeloperoxidase	279.42	x	95,971.49	8,1071.72	x
Prostaglandin-endoperoxide synthase 1	76.84	15.96	103.93	19.80	0.33

Prostaglandin-endoperoxide synthase 2	4.63	1.22	15.08	5.11	0.09

Recombination activating gene 2	10334.28	x	x	x	x
Serine (or cysteine) peptidase inhibitor, clade B, member 1b	721.21	169.42	979.14	436.54	0.60
**Oxygen transporters**					
Cytoglobin	21.19	8.44	35.98	11.78	0.37
Dynamin 2	39.75	9.21	45.30	4.03	0.60
Fanconi anemia, complementation group C	62.37	12.39	66.50	8.71	0.79
Hemoglobin alpha, adult chain 2	293.67	221.16	88.18	36.69	0.39
Intraflagellar transport 172 homolog (*Chlamydomonas*)	14.65	4.23	26.25	6.62	0.19
Myoglobin	2,014.18	1,400.79	1,021.15	101.09	0.58
Neuroglobin	147.26	24.94	128.38	34.92	0.68
Solute carrier family 38, member 1	2.26	0.13	3.07	0.72	0.31
Solute carrier family 38, member 5	99.09	14.70	82.06	18.44	0.50
Vimentin	9.04	4.64	8.84	5.53	0.98
**Other**					
Similar to serine/threonine-protein kinase ATR (Ataxia telangiectasia and Rad3-related protein)	21,362.89	9,743.84	14,220.24	5,207.04	0.59
Selenoprotein S	4.82	0.67	5.62	0.93	0.51

*All relative gene expression from implanted animals compared to no surgery control animals. The bold lines indicate genes that were expressed with statistical significance p < 0.05. The dashed lines indicate the genes that were near statistical significance p = 0.06–0.09. Power analysis revealed that a sample size of 9 ± 1 animals per group, would obtain statistical significance with genes indicating a p = 0.06–0.09*.

### Oxidative Stress Histological Markers after Electrode Implantation

**Figure 3:**
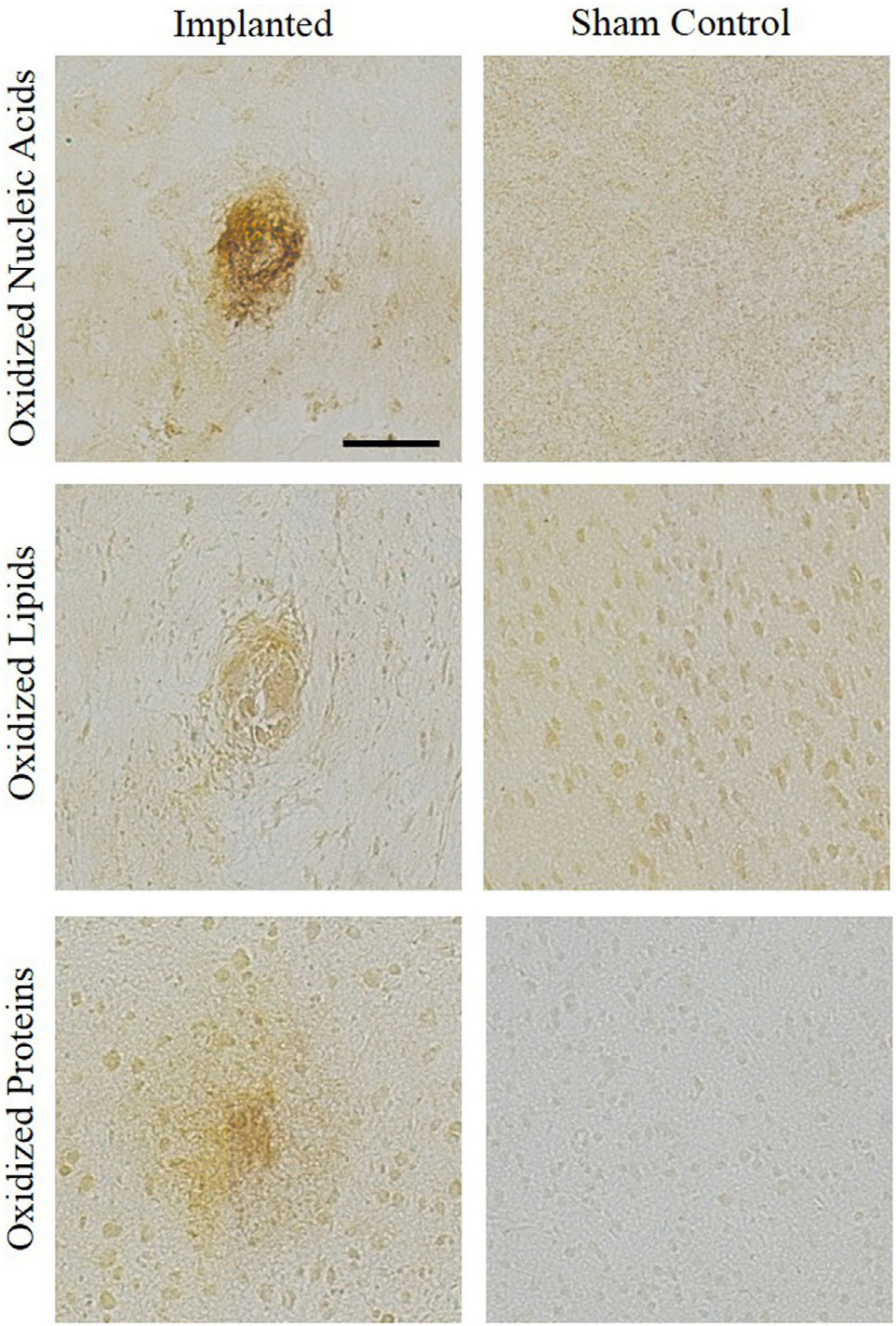
Oxidative stress histological markers. An accumulation of oxidative stress markers around the implant site were shown through staining for hydroxydeoxygaunosine (oxidized nucleic acids), hydroxynonenal (oxidized lipids), and nitrotyrosine (oxidized proteins). No surgery sham controls were stained for comparison.

Representative images showing a presence of oxidative stress markers for nucleic acid, lipid, and protein damage around the area of implantation are compared to sham control stained tissue (Figure 3). The qualitative images demonstrate increased levels of oxidative damage around the implant site. The images in Figure 3 were stained for hydroxydeoxygaunosine (8-OHdG), a marker of oxidized nucleic acids (Wu et al., 2004), hydroxynonenal (HNE), a marker of oxidized lipids (Ihara et al., 1999), and NT, a marker of oxidized proteins, respectively (Sun et al., 2002). These images clearly show that the there is an accumulation of oxidative stress markers surrounding the site of intracortical microelectrode implantation.

## Discussion

Oxidative stress has been common link between neurological injuries and neurodegenerative disorders (Cobb and Cole, 2015; Kim et al., 2015). However, the presence of oxidative stress following intracortical microelectrode implantation is not clearly defined. Therefore, it was the goal of this study to investigate the presence of oxidative stress after intracortical microelectrode implantation, through gene expression and histological markers. The results of this study have shown a direct connection of oxidative stress markers to intracortical microelectrode implantation. Gene expression analysis revealed four genes to be significantly different in animals implanted with intracortical microelectrodes compared to no surgery control animals. The genes that were significantly overexpressed in animals receiving surgery each play a different, but important role in the physiology of the brain tissue. However, these precise genes are not directly connected within one specific pathway. Therefore, the mechanism underlying oxidative stress following intracortical microelectrode implantation is not yet fully understood. However, this study illustrates imperative, novel insight on the oxidative stress response to implanted intracortical microelectrodes.

The significant increase in Ehd2 gene expression in microelectrode implanted animals aligns with the neuroinflammatory response. EH domains are protein interaction molecules that are associated with the functions of regulating intracellular protein transport/sorting and membrane trafficking, as well as with endocytosis (Carbone et al., 1997; Salcini et al., 1997; Mayer, 1999). The function of Ehd2 in central nervous system diseases is still incomplete. Ke et al. (2014) investigated the Ehd2 expression in adult rats after intracerebral hemorrhage (a subtype of stroke) and found Ehd2 was upregulated in the perihematomal caudate. Furthermore, Ke et al. (2014) found that Ehd2 was co-localized with apoptotic neurons and activated microglia after intracerebral stroke. A hallmark of the neuroinflammatory response observed after intracortical microelectrode implantation includes activated microglia and a neuronal dieback around the microelectrode interface (Polikov et al., 2005; Jorfi et al., 2014). The role of activated microglia in the neuroinflammatory response is to phagocytose the foreign body (i.e., microelectrode). Thus, the increased expression of Ehd2 was consistent with the validated and understood neuroinflammatory response to implanted microelectrodes.

In adults, the neurons in the brain and spinal cord highly express prion proteins, while glial cells (i.e., astrocytes, microglia, and oligodendrocytes) in the central nervous system and some peripheral nervous system cells (i.e., axons and Schwann cells) express prion proteins at lower levels (Moser et al., 1995; Ford et al., 2002; Westergard et al., 2007; Ding et al., 2013). The prion protein has been shown to be involved in cell death and survival, oxidative stress, immunomodulation, differentiation, metal ion trafficking, cell adhesion, and transmembrane signaling (Aguzzi et al., 2008; Linden et al., 2008). Several neurodegenerative pathologies have been associated with the misfolding of prion proteins, including Alzheimer’s disease and Parkinson’s disease (Wemheuer et al., 2017).

Alternatively, there has also been evidence suggesting that prion proteins may protect cells from oxidative stress (Milhavet and Lehmann, 2002; Westergard et al., 2007). For example, cell culture studies utilizing neurons from Prnp^−/−^ mice were more susceptible to oxidative stress compared to neurons cultured from wild-type (WT) mice (Brown et al., 1997, 2002). Furthermore, brain tissue from the Prnp^−/−^ mice had higher levels of protein oxidation and lipid peroxidation compared to WT mice of the same genetic background (Wong et al., 2001). Accordingly, it is feasible to hypothesize that the increase in prion protein gene expression observed here is in response to the oxidative stress, and the prion protein gene expression is playing a neuroprotective role.

Chronic blood–brain barrier breach has been shown to correlate with increased neuroinflammation and a reduction in intracortical microelectrode performance (Kozai et al., 2010, 2015; Saxena et al., 2013). The significant differences of Scd1 and Noxa1 gene expression, both genes typically found in the systemic and cerebral vasculature, lead us to hypothesize the breaching of the blood–brain barrier after microelectrode implantation could be an initiator of the observed increase in oxidative stress. Scd1 catalyzes the synthesis of monounsaturated fatty acids, palmitoleate, and oleate, from saturated fatty acids, palmitate, and stearate, respectively (Ntambi and Miyazaki, 2003; Flowers and Ntambi, 2008; Igal, 2010; Ralston et al., 2016). The regulation of Scd1 expression has been shown to effect the inflammatory response in various cell and tissue types, including adipocyte and macrophage inflammation (Liu et al., 2011). Uryu et al. (2003) found that when inflammation induced by β-amyloid peptide activation of macrophage occurred, Scd1 gene expression was significantly upregulated, as well as, a set of pro-inflammatory genes. In a later clinical study by Astarita et al. (2011), it was found that the gene expression of Scd1 was significantly elevated in patients with Alzhemier’s disease, thus connecting the presence of Scd1 in a diseased brain.

Nox enzymes are transmembrane carriers that reduce oxygen to superoxide anion by transporting electrons from cytosolic NADPH in tissues throughout the body (Ma et al., 2017). Specifically, Nox1 has been found in various areas around the brain, including the cerebral cortex, hippocampus, cerebellum, substantia nigra, striatum, hypothalamus, and cerebral vessels (Hernandes and Britto, 2012; Ma et al., 2017). Several Nox enzymes have been linked to neurodegenerative disorders and injuries. Relevant to this study, Nox1 has been studied in stroke, Parkinson’s disease and ALS disease models (Marden et al., 2007; Kahles et al., 2010; Cristóvão et al., 2012). Interestingly, many TBI studies have noted Nox2 activation following cortical injury as early as 1 h and up to 28 days post-TBI (Zhang et al., 2012; Cooney et al., 2013; Lu et al., 2014). However, studies evaluating traumatic brain injury due to cortical impact have not examined the activation of Nox1enzymes. Nox2 expression is highly associated with activated microglia ROS production (Brown and Griendling, 2009; Hernandes and Britto, 2012). Other Nox isoforms have been reported to be elevated in the cortex after TBI, including Nox3 and Nox4. Nox3 was also shown to be present in both injured and uninjured neurons (Cooney et al., 2013). As far as we know, this study is the first to investigate the gene expression of Noxa1 after intracortical microelectrode implantation, or any neurological injury for that matter.

In order to verify the gene expression results, histological staining of oxidative stress markers was performed on the adjacent tissue from the same animal. Previous research has shown increased levels of the oxidative stress markers NT, HNE, and 8-OHdG in neural diseases and disorders. For example, Kuhn et al. (2004) showed that elevated levels of NT correlated to neuronal toxicity leading to the death of dopaminergic neurons. Additionally, Kruman et al. showed that elevated HNE levels led to neuronal apoptosis; while Gmitterová et al. (2009) showed that Parkinson’s patients had elevated levels of 8-OHdG in the cerebrospinal fluid. Therefore, the positive histological staining for modified lipids, nucleic acids, and proteins adjacent to the site of intracortical microelectrode implantation indicates that there is a direct correlation between oxidative stress and intracortical microelectrode implantation. While previous studies have shown the link between neurodegenerative disease and oxidative stress (Chen et al., 2012), the current study links intracortical microelectrode implantation with the presence of both histological markers of oxidative stress and changes in gene regulation characteristic of increased oxidative stress.

Oxidative stress plays a role in the inflammatory response, recording quality and failure of the electrodes. Although antioxidants have shown some potential to mitigate this response, they target various pathways and some quench ROS entirely (Potter et al., 2013, 2014; Potter-Baker et al., 2015; Nguyen et al., 2016). We do not want to inhibit all of these pathways and eliminate all ROS production, as that will encumber wound healing and normal physiological processes (Popa-Wagner et al., 2013). Here we have identified four genes of interest that can be targeted for therapies. Therefore, when developing new therapeutic treatment strategies to mitigate the oxidative stress and inflammation around implanted microelectrodes, we envision the utilization of successful strategies accomplished by the cancer research community with targeted gene therapy. RNA interference (RNAi) targeted gene therapy has been employed in novel cancer treatments (ex HER2+) (Gavrilov and Saltzman, 2012; Mansoori et al., 2014; Ahmed et al., 2015). We further envision the utilization of RNAi mechanisms, as well as gene knock out models, in order to validate the role of specific genes with oxidative stress following intracortical microelectrode implantation. Following which, RNAi-based drugs can be used as a therapy to reduce oxidative stress around implanted probes.

## Conclusion

Together, gene expression and histological staining demonstrated oxidative damage at the intracortical microelectrode/tissue interface at 4 weeks postimplantation. The increased gene expression of Ehd2, Prnp, and Scd1 along with the positive staining for oxidized proteins, lipids, and nucleic acids revealed an increase in oxidative stress around the implant site compared to the no surgery control animals. This study shows the first direct evidence of oxidative stress following microelectrode implantation and lays the foundation for more detailed mechanistic studies to come. Through the quantitative measurement of these and other genes associated with oxidative damage, at all stages of neuroinflammation and neurodegeneration following intracortical microelectrode implantation, future studies can identify therapeutic targets to mitigate deleterious protein, lipid, and nucleic acid modifications due to oxidative stress pathways associated with microelectrode implantation, including the use of small interfering RNA-mediated gene silencing for specific genes identified in the current study.

## Ethics Statement

All animal procedures were approved by the Institutional Animal Care and Use Committee (IACUC) at the Louis Stokes Cleveland Department of Veterans Affairs Medical Center.

## Author Contributions

EE and JC contributed substantially to the conception or design of the work, analysis, and interpretation of data for the work, drafting, and revising the manuscript for important intellectual content, approved the final version to be published, and agreed to be accountable for all aspects of the work. GR and JH performed the histology experiments and drafted corresponding sections of the manuscript. CS, SM, JR, KC, and HF helped with the acquisition of gene expression data and initial drafts of the manuscript. All authors (EE, GR, JH, CS, SM, JR, KC, HF, and JC) approved the final version to be published and agreed to be accountable for all aspects of the work.

## Conflict of Interest Statement

The authors declare that the research was conducted in the absence of any commercial or financial relationships that could be construed as a potential conflict of interest.
